# Recurrent diffuse lung disease due to surfactant protein C deficiency

**DOI:** 10.1016/j.rmcr.2018.07.003

**Published:** 2018-07-19

**Authors:** Brigitte Kazzi, David Lederer, Emilio Arteaga-Solis, Anjali Saqi, Wendy K. Chung

**Affiliations:** a College of Physicians and Surgeons, Columbia University, USA; b Department of Medicine, Columbia University Medical Center, USA; c Department of Epidemiology, Columbia University Medical Center, USA; d Department of Pathology & Cell Biology, Columbia University Medical Center, USA; e Department of Pediatrics, Columbia University Medical Center, USA

**Keywords:** Childhood diffuse lung disease, Hydroxychloroquine, Surfactant protein C deficiency

## Abstract

Surfactant protein C (SP-C) deficiency causes diffuse lung disease with variable prognosis and severity that usually presents in infancy. We present the case of a patient with diffuse lung disease who was successfully treated with hydroxychloroquine and steroids in infancy, who presented again as a young adult with respiratory symptoms. Exome sequencing identified a novel *de novo SFTPC* mutation (c.397A > C p.S133R). Mutated SP-C accumulates and leads to injury of alveolar type II cells, which normally replenish alveolar type I cells after injury. This may explain the symptom recurrence after lung injury in young adulthood. Although hydroxychloroquine has been hypothesized to interfere with mutated SP-C accumulation, data on long term outcome remains limited.

## 1 Introduction

Children's diffuse lung diseases (DLD) encompass a heterogeneous group of conditions defined by at least three of the following: 1-respiratory symptoms; 2-respiratory signs; 3- hypoxemia; and 4-diffuse abnormalities on chest imaging [1]. Some resemble adult interstitial lung diseases, but in infancy (<2 yr old) many DLDs, such as neuroendocrine hyperplasia and pulmonary interstitial glycogenosis, are not seen in adults. The severity and prognosis of DLD in infancy varies, ranging from mild symptoms with supplemental oxygen requirement for pulmonary interstitial glycogenosis to poor outcomes with extensive fibrosis for surfactant protein B mutations [2].

Mutations in several surfactant-related genes have been increasingly recognized as causes of DLD in infants. Surfactant is composed of phospholipids and proteins secreted by alveolar type II cells (AEC2s). Surfactant protein C (SP-C) normally helps reduce surfactant tension at the air liquid interface, has anti-inflammatory properties, and signals cell differentiation [3]. Mutated SP-C accumulates and leads to injury of AEC2s, which normally replenish alveolar type I cells (AEC1s) after injury. Because of the low prevalence of DLD in infancy, evidence-based treatments are limited [4]. We report a case of DLD due to a novel *de novo* Surfactant Protein C (*SFTPC)* mutation successfully treated with hydroxychloroquine and prednisone in infancy followed by recurrence in young adulthood.

## 2 Case report

The full-term male patient had a neonatal period notable for a wet cough. After discontinuing breastfeeding at four months of age, he had inadequate weight-gain and his wet cough became severe, especially at night. He was tachypneic with no perceptible wheezing. At 9-months, chest-x-ray and CT were normal. At 11-months, he was admitted for respiratory distress with severe hypoxemia, cyanosis, and clubbing. A chest x-ray showed diffuse interstitial markings on both lung fields and a chest CT revealed extensive interstitial markings. The Tc-DTPA half-time clearance was 13 minutes bilaterally, indicating increased pulmonary epithelial permeability. A sweat test was normal. Lung biopsy demonstrated diffuse moderate interstitial fibrosis with eosinophilic inflammation and alveolar epithelial cell hyperplasia. Parainfluenza was recovered from the cultured lung biopsy tissue. He was diagnosed with interstitial lung disease and treated with prednisone and hydroxychloroquine. He required oxygen supplementation for three months. At 3-years, his pharmacological therapy was stopped since his symptoms had resolved, and his pulmonary function and chest X-ray had normalized. He grew and functioned normally for the next 24 years, including participating in sports and living at altitudes ranging from 6000 to 12,000 feet with no dyspnea.

**Fig. 1:**
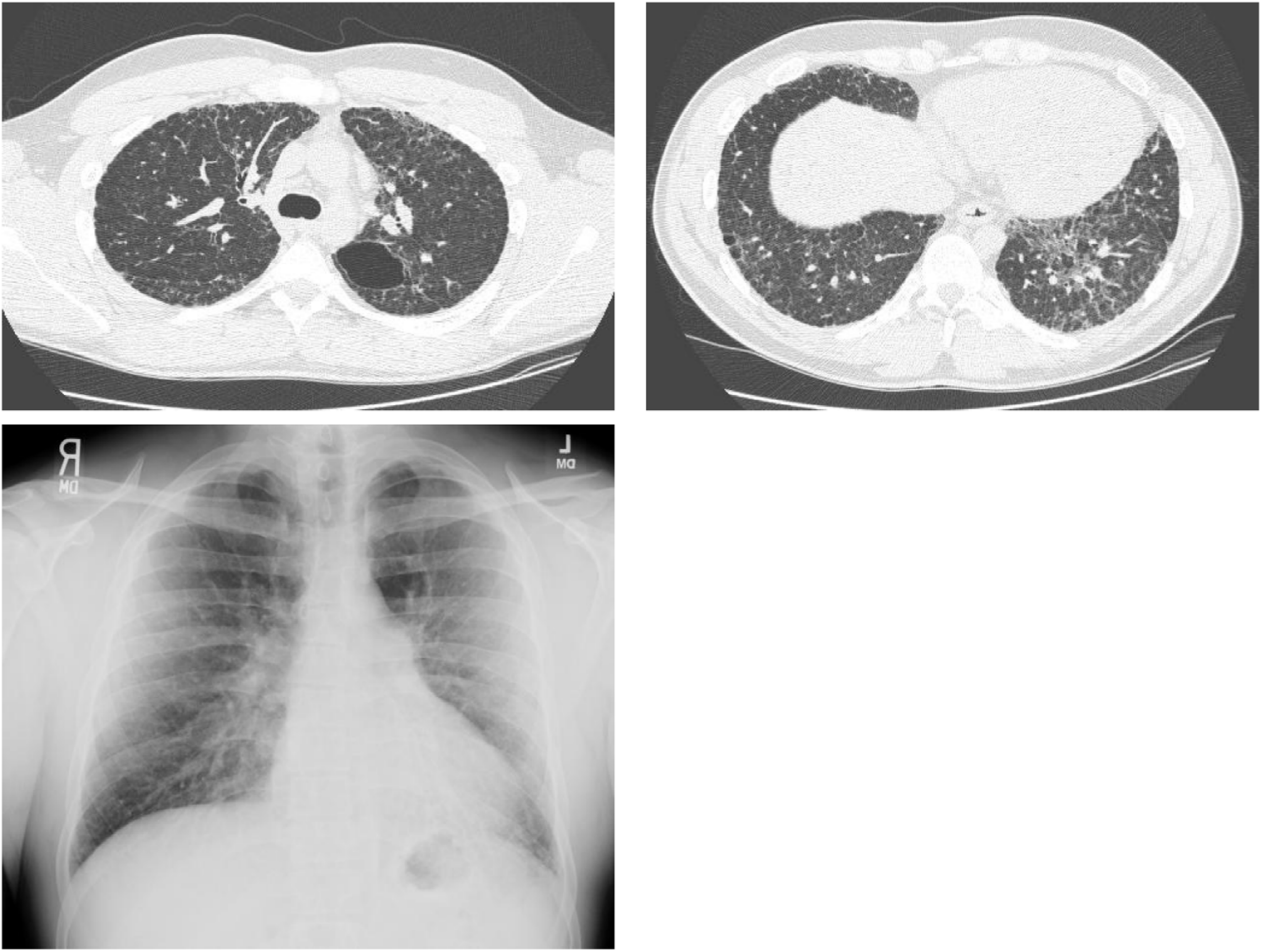
Chest CT and X-rays, patient age 27 years.

At 27-years of age, he again developed a dry cough and dyspnea during strenuous activities. He reported that a leaky shower had led to water damage on his bedroom wall the year prior. Air samples from the affected area of the home grew Aspergillus/Penicillum-like mold. His Aspergillus antibody and precipitin test were negative. A review of other environmental exposures and autoimmune symptoms was unrevealing. There was no known family history of lung disease. His physical examination was notable only for bilateral crackles heard posteriorly. There was no clubbing. Forced vital capacity was 68% predicted, FEV_1_ was 65% predicted, the FEV_1_/FVC ratio was 0.79, and the diffusing capacity was 48% predicted. A high-resolution computed tomographic scan of the chest showed diffuse interlobular septal thickening with several areas of bulla or large cysts, without bronchiectasis or significant fibrosis (Fig. 1).

**Fig. 2:**
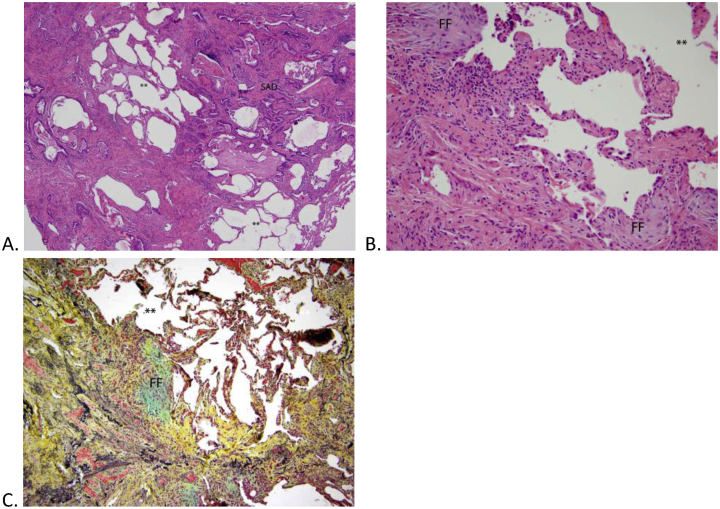
A: Low magnification of the lower lobe shows temporal heterogeneity with dense pink areas representing fibrosis, intervening uninvolved areas (**) and small airways disease (SAD) (top right). B: High magnification demonstrates fibrosis (left lower) and relatively uninvolved areas (**) with fibroblastic foci (FF) separating the two. C: A high magnification on the Movat stain highlights the fibroblastic foci (FF) alongside fibrosis (yellow) and uninvolved lung (**). (For interpretation of the references to colour in this figure legend, the reader is referred to the Web version of this article.)

A right sided thoracoscopic lung biopsy showed patchy, fibrosing and minimally cellular interstitial pneumonitis, focal pleural fibrosis and adhesions on the right upper and middle lobes. Biopsy of the right lower lobe showed fibrosing and cellular interstitial pneumonitis with extensive peribronchiolar metaplasia, pleuritis, adhesions, and extensive fatty metaplasia (Fig. 2). Some metaplasia was also present in the upper and middle lobes (data not shown).

Exome sequencing identified a heterozygous novel *de novo* c.397A > C p.S133R, likely pathogenic variant in *SFTPC*, consistent with a diagnosis of surfactant metabolism dysfunction (OMIM 178620). Surfactant protein C, which is expressed as a pro-protein in AEC2s, is critical for surfactant homeostasis. Mutations in *SFTPC* result in the production of an abnormal pro-protein that accumulates in AEC2s [5]. Chloroquine or hydroxychloroquine has been previously reported to improve symptoms in patients with surfactant protein C dysfunction [6]. Hydroxychloroquine 200mg twice daily was added to his regimen, and his prednisone dose has been tapered to 15mg daily over the subsequent three months. Five months after initiating prednisone, his FVC was 76% predicted and his DLCO was 58% predicted.

## 3 Discussion

SP-C deficiency is a rare lung disease with highly variable age of onset, severity and natural history (Table 1).

**Table 1:** Mutations previously observed in *SFTPC* and associated clinical phenotype.

Mutation	Age of onset	Severity	Response to treatment	Reference
IVS4DS, G-A, +1	Mother diagnosed at 1 year	Respiratory insufficiency Died after childbirth	Treated with glucocorticoids until 15 yo	Nogee et al. [27]
	Son diagnosed in infancy	Respiratory insufficiency	NA	
p.ILE38PHE c.112 A > T	Diagnosed at 7 months; in infancy; unknown age of onset of brother	Recurrent pneumothorax, repetitive cough, hypoxia, failure to thrive, and gastro-esophageal reflux	Treated for 38 months - asymptomatic	Avital et al. [6]
p.VAL39LEU c.115 G > T	11 years old	Cough, shortness of breath on minimal exertion at 11 yo	Significant improvement after 1 week of treatment – treated for 18 months - asymptomatic	Avital et al. [6]
	NA	Asymptomatic mother	NA	
VAL39ALA c.116 T > C	4 months Virus infection at onset	ILD	NA	Guillot et al. [28]
GLU66LYS 1509 G-A	13 days	Respiratory distress	Tracheotomy, mild ventilatory support, supplemental O2	Stevens et al. [29]
ILE73THR c.218 T > C	5.9 months (average for 10 patients) not neonatal 1 month - 2.5 years	Variable (asymptomatic to fatal)	Patient treated at 2 months: improved within 5 weeks of treatment – treated for 7 months – restarted at 19 yo for 6 years Patient treated at 16 months for 7 months, restarted 6 weeks later for 7 months – cough in adolescence and adulthood, SOB during exercise Patient treated at 5.5 years (symptomatic at 2.5) for 16 months with good response - asymptomatic	Avital et al. [6]
-- c.325-1 G > A	Birth Viral infection at onset	NA	NA	Guillot et al. [28]
p.TYR113HIS c.337 T > T/C	7 days	Cough, tachypnea and exhibited failure to thrive	Slight improvement on antibiotics and nasal oxygen	Da Hong et al. [30]
ALA116ASP	5 months	Respiratory insufficiency	Good response to hydroxychloroquine	Rosen and Waltz [31]
p.HIS142fs c.424delC	Birth Viral infection at onset	NA	NA	Guillot et al. [28]
GLN145HIS c.435 G > C	1 month	NA	NA	Guillot et al. [28]
ARG167GLN g.2125 G > A	9 months	-respiratory insufficiency, failure to thrive, anemia -Fatal at 18 months after chronic symptoms-Asymptomatic	Repetitive therapeutic broncho-alveolar lavages	Tredano et al. [32]
LEU188GLN	Variable	Variable (adults with usual interstitial pneumonitis and children with cellular nonspecific interstitial pneumonitis)	NA	Thomas et al. [33]
LEU188PRO c.563 T > C	2 months	NA	NA	Guillot et al. [28]
LEU188GLN c.563 T > A	1 day	Respiratory failure, decannulated from chronic ventilation at 5 years	Clearance, steroids, azathioprine	Liptzin et al. [34]
CYS189TYR c.566 G > A	Birth No viral infection in n = 2, others unknown	Variable: severe in first months of life and asymptomatic in adults		Guillot et al. [28]
p.CYS189TRP c.567 C > G	4 months	Tachypnea in infancy	Steroids, azithromycin, hydroxychloroquine 8 months to 3 years on home ventilation	Liptzin et al. [34]
LEU194PRO 581T-C	2 months (virus infection at onset) and adult	Infant with ILD – Adults with pulmonary fibrosis	NA	Guillot et al. [28]

It is associated with acute respiratory failure and interstitial lung disease [7] and has been described in neonates with severe, fatal disease as well as in adults who remain asymptomatic for years [[8], [9], [10], [11], [12]]. The variability is likely due to the type of mutation, age, modifier genes, and treatment [13]. Our patient represents one of the few cases of SP-C deficiency with long term outcome after initial hydroxychloroquine and steroid treatment for infantile DLD. Patients with *SFTPC* mutations and DLD respond to hydroxychloroquine, generally with elimination of pulmonary symptoms and without limitations in daily life [4,5]. Treatment has been stopped after variable periods of time (a few months [14] to seven years [8]) and restarted in cases of pulmonary exacerbations [6].

The exact mechanism of action of hydroxychloroquine has not been fully elucidated [15]. When the *SP-C* gene is mutated, the precursor of surfactant protein C (proSP-C) is misfolded and accumulates within the ER and Golgi apparatus of AEC2s, leading to cellular injury and apoptosis [6]. It has been suggested that the lung disease is not only due to the lack of normal SP-C but also to the expression of abnormal proSP-C [16]. Chloroquine and hydroxychloroquine may affect lysosomal activities to decrease vesicle fusion, decrease exocytosis, and decrease digestive efficiency of phagolysosomes [[17], [18], [19]], interfering with abnormal proSP-C accumulation [8] and its intracellular post-translational processing [15].

SFTPC-positive AEC2s are long-term tissue stem cells that can self-renew and differentiate into AEC1s for over a year to replenish alveolar lining after injury or viral infection [20]. SP-C is one of the gene products that signals the onset of cell differentiation [21]. In other SP-C deficient patients, symptom recurrence has been associated with viral infection or lung injury: diffuse lung injury triggers the destruction of AEC1s and stimulates the proliferation of AEC2s to restore the alveolar epithelium. However, it is plausible that apoptosis of AEC2s [6] and inhibition of AEC2 proliferation in patients with an SFTPC mutation leads to pulmonary fibrosis [22]. In SP-C deficient mice (*Sftpc−/−*) in a 129S6 background, which develop spontaneous interstitial lung disease with age [23], intra tracheal *Pseudomonas aeruginosa* leads to an exaggerated inflammatory response and bacterial load resulting in decreased survival [23]. Additionally, *Sftpc−/−* mice have an increased and persistent inflammation, and pulmonary fibrosis in response to LPS and bleomycin induced lung injury, respectively [24,25]. Whereas, restoration of SP-C in a *Sftpc−/−* compound transgenic mouse protected the mice against respiratory syncytial virus induced inflammation and improved viral clearance [26]. These findings suggest that in addition to SP-C being important in maintaining the bio-physical properties of surfactant, it plays an important role in limiting pulmonary inflammatory responses to injury.

## 4 Conclusion

The use of hydroxychloroquine in our patient for two years early in life might have inhibited the accumulation of misfolded proSP-C and AEC2 damage in infancy, enabling normal lung growth and maturation. Recurrence and worsening of the symptoms 24 years after hydroxychloroquine discontinuation may be related to progressive accumulation of proSP-C in AEC2 over the intervening 24 years after an infection exacerbated lung injury by impairing AEC2 proliferation and differentiation.

Successful reports of treatment of children with *SFTPC* mutations with hydroxychloroquine demonstrates the clinical utility of genetic testing for children with DLD.
